# Supplementary material for: Assessment of Concentration KRT6 Proteins in Tumor and Matching Surgical Margin from Patients with Head and Neck Squamous Cell Carcinoma
Source: Int J Mol Sci. 2024 Jul 4;25(13):7356. doi: 10.3390/ijms25137356 (PMC11242288; doi:10.3390/ijms25137356)
Supplement: Supplementary file 1 [file ijms-25-07356-s001.zip › ijms-3046592-supplementary.pdf]

Table S1

Parameter	OSCC	OPSCC+HPSCC+LSCC	p-value; OR (95% CI)
Age (mean±SD)	62.73±11.29	65.11±11.46	p=0.480;
Sex			
female	13	2	p=0.0203;<br>6.882 (1.349-35.114)
male	17	18	
Smoking			
yes	27	17	p=0.596;<br>1.588 (0.287-8.795)
no	3	3	
Drinking			
yes occasionally	22	11	p=0.184;<br>2.250 (0.680-7.442)
yes regularly	8	7	p=0.530;<br>0.675 (0.199-2.298)
no	0	2	p=0.181;<br>0.121 (0.006-2.668)
Clinical T- classification (T)			
T1	2	1	p=0.786;<br>1.407 (0.119-16.659)
T2	5	5	p=0.510;<br>0.625 (0.155-2.528)
T3	11	7	p=0.834;<br>1.135 (0.347-3.716)
T4	11	7	p=0.834;<br>1.135 (0.347-3.716)
NA	1	0	-
Nodal status (N)			
N0	20	8	p=0.069;<br>3.056 (0.917-10.180)
N1	1	5	**p=0.044;<br>0.100 (0.011-0.940)**
N2	6	6	p=0.397;<br>0.565 (0.151-2.116)
N3	2	0	p=0.422;<br>3.546 (0.161-78.025)
NA	1	1	-
Histological grading (G)			
G1	12	3	p=0.068;<br>3.778 (0.906-15.760)
G2	15	15	p=0.082;<br>0.333 (0.097-1.151)
G3	3	2	P=1.000;<br>1.000 (0.152-6.593)
G4	0	0	P=0.844;<br>0.672 (0.013-35.250)

OR- odds ratio; 95% CI - 95% confidence interval; NA – not assessed.

Table S2

Parameter	The floor of the mouth (F)	The tongue (T)	The jaw (J)	The larynx (L)	p-value; OR (95% CI)
Age (median (range))	58.50 (51-65)	56.50 (51-72)	74 (57.50-85.25)	68 (60-75.50)	F vs. T: p=0.705;<br>F vs. J: p=0.079;<br>F vs. L: p=0.083;<br>T vs. J: p=0.167;<br>T vs. L: p=0.229;<br>J vs. L: p=0.619;
Sex					
female	7	2	3	2	F: p=0.063; 3.571 (0.934-13.662)<br>T: p=0.424; 0.500 (0.092-2.730)<br>J: p=0.150; 4.091 (0.601-27.847)<br>L: p=0.0478; 0.189 (0.036-0.984)
male	7	8	2	15	
Smoking					
yes	13	9	3	14	F: p=0.332; 3.000 (0.326-27.602)<br>T: p=0.608; 1.800 (0.191-16.980)<br>J: p=0.128; 0.208 (0.028-1.569)<br>L: p=0.726; 0.747 (0.146-3.825)
no	1	1	2	3	
Drinking					
yes occasionally	8	8	5	8	F: p=0.584; 0.698 (0.193-2.526)<br>T: p=0.222; 2.857 (0.528-15.411)<br>J: p=0.172; 7.857 (0.407-151.535)<br>L: p=0.090; 0.339 (0.097-1.186)
yes regularly	6	2	0	7	F: p=0.330; 1.917 (0.518-7.099)<br>T: p=0.345; 0.442 (0.081-2.402)<br>J: p=0.218; 0.155 (0.008-3.006)<br>L: p=0.345; 1.838 (0.520-6.500)
no	0	0	0	2	F: p=0.584; 0.421 (0.019-9.340)<br>T: p=0.792; 0.657 (0.029-14.793)<br>J: p=0.823; 1.436 (0.061-34.017)<br>L: p=0.154; 9.516 (0.430-210.827)
Clinical T-classification (T)					
T1	0	0	0	0	F: p=0.665; 2.407 (0.045-127.688)<br>T: p=0.549; 3.381 (0.063-180.940)<br>J: p=0.330; 7.364 (0.132-410.128)<br>L: p=0.810; 1.629 (0.031-85.853)
T2	3	3	0	4	F: p=0.930; 1.071 (0.230-4.991)<br>T: p=0.505; 1.714 (0.351-8.373)<br>J: p=0.381; 0.264 (0.013-5.193)<br>L: p=0.870; 1.128 (0.268-4.757)
T3	5	5	2	6	F: p=0.893; 0.914 (0.244-3.424)<br>T: p=0.467; 1.692 (0.410-6.978)<br>J: p=1.000; 1.000 (0.150-6.670)<br>L: p= 0.616; 0.727 (0.209-2.526)
T4	5	2	3	7	F: p=0.952; 1.042 (0.276-3.927)<br>T: p=0.202; 0.333 (0.062-1.803)

					J: p=0.292; 2.786 (0.415-18.691)<br>L: p=0.714; 1.260 (0.366-4.342)
NA	1	0	0	0	-
Nodal status (N)					
N0	10	5	4	7	F: p=0.129; 3.125 (0.719-13.591)<br>T: p=0.508; 0.619 (0.150-2.560)<br>J: p=0.332; 3.091 (0.316-30.247)<br>L: p=0.122; 0.368 (0.104-1.308)
N1	0	1	0	5	F: p=0.200; 0.145 (0.008-2.779)<br>T: p=0.705; 0.644 (0.066-6.260)<br>J: p=0.622; 0.469 (0.023-9.548)<br>L: p=0.0296; 12.273 (1.282-117.450)
N2	2	4	0	4	F: p=0.457; 0.523 (0.095-2.884)<br>T: p=0.149; 3.111 (0.666-14.543)<br>J: p=0.369; 0.255 (0.013-5.026)<br>L: p=0.786; 1.222 (0.287-5.198)
N3	1	0	1	0	F: p=0.529; 2.500 (0.144-43.287)<br>T: p=0.763; 0.619 (0.028-13.951)<br>J: p=0.136; 9.500 (0.494-182.808)<br>L: p=0.473; 0.321 (0.015-7.116)
NA	1	0	0	1	-
Histological grading (G)					
G1	6	3	2	3	F: p=0.231; 2.250 (0.597-8.481)<br>T: p=0.973; 0.974 (0.212-4.486)<br>J: p=0.625; 1.611 (0.238-10.897)<br>L: p=0.158; 0.351 (0.082-1.503)
G2	7	6	2	12	F: p=0.430; 0.600 (0.169-2.134)<br>T: p=0.925; 1.071 (0.257-4.469)<br>J: p=0.379; 0.427 (0.064-2.842)<br>L: p=0.214; 2.240 (0.628-7.995)
G3	1	1	0	2	F: p=0.805; 0.744 (0.071-7.843)<br>T: p=0.869; 1.222 (0.113-13.209)<br>J: p=0.859; 0.758 (0.036-16.089)<br>L: p=0.576; 1.800 (0.230-14.110)
G4	0	0	1	0	F: p=0.846; 0.724 (0.028-18.874)<br>T: p=0.943; 1.127 (0.043-29.768)<br>J: p=0.052; 27.667 (0.975-784.818)<br>L: p=0.713; 0.543 (0.021-14.078)

OR- odds ratio; 95% CI - 95% confidence interval; NA – not assessed.

Table S3

Parameter	T1+T2	T3	T4	p-value; OR (95% CI)
Age (median (range))		63<br>(53.50-72)	61.5<br>(52.50-71.50)	T1+T2 vs. T3: p=;<br>T1+T2 vs. T4: p=;<br>T3 vs. T4: p=0.897;
Sex				
female	2	5	10	T1+T2: p=0.140; 0.291 (0.057-1.498)<br>T3: p=0.459; 0.625 (0.180-2.167)<br>T4: p=0.038; 3.571 (1.062-12.010)
male	11	14	10	
Smoking				
yes	12	15	17	T1+T2: p=0.389; 2.625 (0.292-23.640)<br>T3: p=0.395; 0.517 (0.113-2.364)<br>T4: p=0.951; 1.049 (0.222-4.968)
no	1	4	3	
Drinking				
yes occasionally	7	16	12	T1+T2: p=0.238; 0.458 (0.126-1.672)<br>T3: p=0.058; 3.930 (0.956-16.149)<br>T4: p=0.377; 0.587 (0.180-1.912)
yes regularly	5	3	7	T1+T2: p=0.380; 1.813 (0.480-6.844)<br>T3: p=0.125; 0.328 (0.079-1.361)<br>T4: p=0.440; 1.615 (0.478-5.463)
no	1	0	1	T1+T2: p=0.427; 3.167 (0.184-54.571)<br>T3: p=0.473; 0.323 (0.015-7.090)<br>T4: p=0.735; 1.632 (0.096-27.650)
Nodal status (N)				
N0	9	11	11	T1+T2: p=0.256; 2.318 (0.543-9.901)<br>T3: p=0.745; 0.825 (0.259-2.627)<br>T4: p=0.498; 0.672 (0.213-2.118)
N1	0	2	4	T1+T2: p= 0.294; 0.206 (0.011-3.934)<br>T3: p=0.833; 0.824 (0.136-4.988)<br>T4: p=0.162; 3.625 (0.597-22.014)
N2	3	6	3	T1+T2: p= 0.891; 1.111 (0.247-5.000)<br>T3: p=0.301; 2.000 (0.538-7.434)<br>T4: p=0.256; 0.431 (0.101-1.843)
N3	0	0	2	T1+T2: p=0.747; 0.600 (0.027-13.361)<br>T3: p=0.461; 0.313 (0.014-6.869)<br>T4: p=0.174; 8.514 (0.387-187.143)
NA	1	0	0	-
Histological grading (G)				
G1	4	7	3	T1+T2: p=0.719; 1.289 (0.324-5.122)<br>T3: p=0.226; 2.167 (0.620-7.571)<br>T4: p=0.135; 0.337 (0.081-1.405)
G2	8	10	14	T1+T2: p=1.000; 1.000 (0.275-3.633)<br>T3: p=0.319; 0.556 (0.175-1.764)<br>T4: p=0.324; 1.815 (0.555-5.931)
G3	1	2	2	T1+T2: p=0.787; 0.729 (0.074-7.181)<br>T3: p=0.866; 1.177 (0.179-7.753)<br>T4: p=0.941; 1.074 (0.163-7.063)
G4	0	0	1	T1+T2: p= 0.976; 0.951 (0.037-24.767)<br>T3: p=0.723; 0.556 (0.022-14.321)<br>T4: p=0.332; 5.000 (0.194-128.891)

OR- odds ratio; 95% CI - 95% confidence interval; NA – not assessed.

Table S4

Parameter	N0	N1	N2+N3	p-value; OR (95% CI)
Age (mean±SD)	66.77±11.75	63.67±10.80	56.17±9.32	N0 vs. N1: p=0.806;<br>N1 vs. N2+N3: p=0.375;<br>**N0 vs. N2+N3: p=0.01958;**
Sex				
female	14	1	2	N0: p=0.033; 4.667 (1.132-19.242)<br>N1: p=0.373; 0.363 (0.039-3.379)<br>N2+N3: p=0.091; 0.244 (0.048-1.253)
male	17	5	12	
Smoking				
yes	27	5	11	N0: p=0.499; 1.688 (0.370-7.697)<br>N1: p=0.944; 0.921 (0.093-9.127)<br>N2+N3: p=0.492; 0.573 (0.117-2.801)
no	4	1	3	
Drinking				
yes occasionally	23	4	8	N0: p=0.289; 1.917 (0.576-6.384)<br>N1: p=0.912; 0.903 (0.148-5.524)<br>N2+N3: p=0.281; 0.494 (0.1367-1.782)
yes regularly	6	2	6	N0: p=0.113; 0.360 (0.102-1.273)<br>N1: p=0.732; 1.375 (0.223-8.499)<br>N2+N3: p=0.137; 2.719 (0.729-10.142)
no	2	0	0	N0: p=0.429; 3.475 (0.158-76.230)<br>N1: p=0.856; 1.339 (0.058-31.119)<br>N2+N3: p=0.651; 0.490 (0.022-10.840)
Clinical T-classification (T)				
T1	2	0	1	N0: p=0.830; 1.310 (0.111-15.479)<br>N1: p=0.965; 0.934 (0.0431 to 20.244)<br>N2+N3: p=0.815; 1.346 (0.112-16.131)
T2	7	0	2	N0: p=0.262; 2.625 (0.486-14.171)<br>N1: p=0.420; 0.296 (0.015-5.725)<br>N2+N3: p=0.699; 0.714 (0.129-3.942)
T3	11	2	6	N0: p=0.745; 0.825 (0.259-2.627)<br>N1: p=0.833; 0.824 (0.136-4.988)<br>N2+N3: p=0.611; 1.385 (0.395-4.859)
T4	11	4	5	N0: p=0.498; 0.672 (0.213-2.118)<br>N1: p=0.162; 3.625 (0.597-22.014)<br>N2+N3: p=0.753; 0.815 (0.228-2.916)
NA	0	0	0	-
Histological grading (G)				
G1	10	0	4	N0: p=0.342; 1.905 (0.504-7.198)<br>N1: p=0.233; 0.167 (0.009-3.170)<br>N2+N3: p=0.912; 1.080 (0.275-4.241)
G2	18	5	8	N0: p=0.621; 0.746 (0.233-2.386)<br>N1: p=0.254; 3.654 (0.394-33.881)<br>N2+N3: p=0.743; 0.812 (0.233-2.832)
G3	2	1	2	N0: p=0.329; 0.391 (0.059-2.579)<br>N1: p=0.554; 2.050 (0.190-22.148)<br>N2+N3: p=0.513; 1.889 (0.281-12.710)
G4	1	0	0	N0: p=0.672; 2.016 (0.078-51.964)<br>N1: p=0.625; 2.282 (0.084-62.169)

				N2+N3: p=0.916; 0.839 (0.032-21.814)

OR- odds ratio; 95% CI - 95% confidence interval; NA – not assessed.

Table S5

Parameter	G1	G2	G3	G4	p-value; OR (95% CI)
Age (mean±SD)	64.13±10.28	62.71±12.60	64.8±7.79	85	G1 vs. G2: p=0.92;<br>G1 vs. G3: p=0.993;<br>G2 vs. G3: p=0.926;
Sex					
female	3	10	4	1	G1: p=0.206; 0.400 (0.097-1.655)<br>G2: p=0.696; 0.796 (0.253-2.502)<br>G3: p=0.087; 4.857 (0.797-29.617)<br>G4: p=0.269; 6.257(0.242-161.536)
male	12	22	2	0	
Smoking					
yes	15	26	4	0	G1: p=0.127; 9.656 (0.527-177.044)<br>G2: p=0.622; 0.684 (0.152-3.088)<br>G3: p=0.262; 0.342 (0.052-2.231)<br>G4: p=0.098; 0.062 (0.002-1.661)
no	0	6	2	1	
Drinking					
yes occasionally	10	21	4	1	G1: p=1.000; 1.000 (0.283-3.537)<br>G2: p=0.845; 0.891 (0.280-2.832)<br>G3: p=1.000; 1.000 (0.165-6.052)<br>G4: p=0.788; 1.563 (0.061-40.305)
yes regularly	5	9	1	0	G1: p=0.573; 1.450 (0.398-5.278)<br>G2: p=0.945; 1.044 (0.310-3.514)<br>G3: p=0.527; 0.486 (0.052-4.542)<br>G4: p=0.910; 0.828 (0.032-21.450)
no	0	2	1	0	G1: p=0.480; 0.336 (0.016-6.909)<br>G2: p=0.789; 1.400 (0.119-16.459)<br>G3: p=0.245; 4.600 (0.351-60.212)<br>G4: p=0.362; 4.810 (0.1640-141.124)
Clinical T-classification (T)					
T1	1	1	1	0	G1: p=0.797; 1.385 (0.116-16.582)<br>G2: p=0.326; 0.290 (0.025-3.432)<br>G3: p=0.195; 5.625 (0.414-76.437)<br>G4: p=0.375; 4.619 (0.157-135.598)
T2	3	7	0	0	G1: p=0.807; 1.208 (0.265-5.508)<br>G2: p=0.543; 1.587 (0.359-7.014)<br>G3: p=0.459; 0.325 (0.017-6.360)<br>G4: p=0.869; 1.318 (0.050-34.716)
T3	7	10	2	0	G1: p=0.226; 2.167 (0.620-7.571)<br>G2: p=0.319; 0.556 (0.175-1.764)<br>G3: p=0.866; 1.177 (0.179-7.753)<br>G4: p=0.723; 0.556 (0.022-14.321)
T4	3	14	2	1	G1: p=0.135; 0.337 (0.081-1.405)<br>G2: p=0.324; 1.815 (0.555-5.931)<br>G3: p=0.941; 1.074 (0.163-7.063)<br>G4: p=0.332; 5.000 (0.194-128.892)
NA	1	0	1	0	-
Nodal status (N)					
N0	10	18	2	1	G1: p=0.342; 1.905 (0.504-7.198)<br>G2: p=0.621; 0.746 (0.233-2.386)

					G3: p=0.329; 0.391 (0.059-2.579)<br>G4: p=0.672; 2.016 (0.078-51.964)
N1	0	5	1	0	G1: p=0.233; 0.167 (0.009-3.170)<br>G2: p=0.254; 3.654 (0.394-33.881)<br>G3: p=0.554; 2.050 (0.190-22.148)<br>G4: p=0.625; 2.282 (0.084-62.169)
N2	4	6	2	0	G1: p=0.603; 1.450 (0.358-5.874)<br>G2: p=0.385; 0.560 (0.152-2.070)<br>G3: p=0.372; 2.400 (0.351-16.395)<br>G4: p=0.987; 1.027 (0.039-26.842)
N3	0	2	0	0	G1: p=0.651; 0.490 (0.022-10.840)<br>G2: p=0.429; 3.475 (0.158-76.230)<br>G3: p=0.766; 1.618 (0.068-38.257)<br>G4: p=0.288; 6.467 (0.207-202.454)
NA	1	1	1	0	-

OR- odds ratio; 95% CI - 95% confidence interval; NA – not assessed.

Table S6

Parameter	Smoking N	No smoking N	p-value; OR (95% CI)
Age (mean±SD)	64.26±10.89	59.63±15.70	p=0.309;
Sex			
female	13	5	p=0.1325;<br>0.325 (0.075-1.406)
male	32	4	
Drinking			
yes occasionally	29	7	p=0.4442;<br>0.518 (0.096-2.795)
yes regularly	15	0	p=0.1265;<br>0.104 (0.006-1.899)
no	1	2	p=0.0498;<br>12.571 (1.002-157.741)
Clinical T- classification (T)			
T1	3	0	p=0.817;<br>1.434 (0.068-30.387)
T2	9	1	p=0.604;<br>1.8 (0.196-16.568)
T3	15	4	p=0.961;<br>1.034 (0.268-4.000)
T4	17	3	p=0.952;<br>1.049 (0.222-4.967)
NA	1	1	-
Nodal status (N)			
N0	27	4	p=0.500;<br>1.688 (0.370-7.697)
N1	5	1	p=0.944;<br>0.921 (0.093-9.127)
N2	9	3	p=0.319;<br>0.441 (0.088-2.205)
N3	2	0	p=0.988;<br>1.024 (0.045-23.304)
NA	2	1	-
Histological grading (G)			
G1	15	0	p=0.127;<br>9.656 (0.527-177.044)
G2	26	6	p=0.622;<br>0.684 (0.152-3.087)
G3	4	2	p=0.262;<br>0.341 (0.052-2.231)
G4	0	1	p=0.109;<br>0.068 (0.0026-1.823)

OR- odds ratio; 95% CI - 95% confidence interval; NA – not assessed.

Table S7

Parameter	Abstinent (A)	Occasional drinker (O)	Regular drinker (R)	p-value; OR (95% CI)
Age (median (range))	79 (71.5-82.75)	66 (58.5-73)	53 (51-65)	A vs. O: p=0.096;<br>O vs. R: p=0.023;<br>**A vs. R: p=0.0069;**
Sex				
female	1	14	3	A: p=1.000; 1.000 (0.085-11.824)<br>O: p=0.227; 2.227 (0.608-8.153)<br>R: p=0.206; 0.400 (0.097-1.655)
male	2	22	12	
Smoking				
yes	1	29	15	A: p=0.0498; 0.079, (0.006-0.998)<br>O: p=0.444; 0.518 (0.096-2.795)<br>R: p=0.127; 9.656 (0.527-177.044)
no	2	7	0	
Clinical T- classification (T)				
T1	0	3	0	A: p=0.544; 2.714 (0.108-68.250)<br>O: p=0.043; 0.259 (0.070-0.958)<br>R: p=0.458; 0.318 (0.016-6.537)
T2	1	4	5	A: p=0.299; 4.556 (0.260-79.883)<br>O: p=0.753; 0.828 (0.255-2.689)<br>R: p=0.111; 3.200 (0.767-13.353)
T3	0	16	3	A: p=0.473; 0.323 (0.015-7.090)<br>O: p=0.058; 3.930 (0.956-16.149)<br>R: p=0.125; 0.328 (0.079-1.361)
T4	1	12	7	A: p=0.735; 1.632 (0.096-27.650)<br>O: p=0.377; 0.587 (0.180-1.912)<br>R: p=0.440; 1.615 (0.478 to 5.463)
NA	1	1	0	-
Nodal status (N)				
N0	2	23	6	A: p=0.429; 3.475 (0.158-76.230)<br>O: p=0.289; 1.917 (0.576-6.384)<br>R: p=0.113; 0.360 (0.102-1.273)
N1	0	4	2	A: p=0.856; 1.339 (0.058-31.119)<br>O: p=0.912; 0.903 (0.148-5.524)<br>R: p=0.732; 1.375 (0.223-8.499)
N2	0	7	5	A: p=0.747; 0.600 (0.027-13.361)<br>O: p=0.383; 0.550 (0.144-2.107)<br>R: p=0.214; 2.381 (0.606-9.353)
N3	0	1	1	A: p=0.427; 3.800 (0.141-102.209)<br>O: p=0.572; 0.441 (0.026-7.533)<br>R: p=0.483; 2.769 (0.161-47.563)
NA	1	1	1	-
Histological grading (G)				
G1	0	10	5	A: p=0.480; 0.336 (0.016-6.909)<br>O: p=1.000; 1.000 (0.283-3.537)<br>R: p=0.573; 1.450 (0.398-5.278)
G2	2	21	9	A: p=0.789; 1.400 (0.119-16.459)<br>O: p=0.845; 0.891 (0.280-2.832)<br>R: p=0.945; 1.044 (0.310-3.514)
G3	1	4	1	A: p=0.245; 4.600 (0.351-60.212)

				O: p=1.000; 1.000 (0.165-6.052)<br>R: p=0.527; 0.486 (0.052-4.542)
G4	0	1	0	A: p=0.362; 4.810 (0.164-141.124)<br>O: p=0.788; 1.563 (0.061-40.305)<br>R: p=0.910; 0.828 (0.032-21.450)

OR- odds ratio; 95% CI - 95% confidence interval; NA – not assessed.

Table S8

Parameter	Ki-67≤20	Ki-67>20	p-value; OR (95% CI)
Age (mean±SD)	67±10.26	63.25±11.78	p=0.465;
Sex			
female	3	14	p=0.456;<br>1.929 (0.343-10.831)
male	3	27	
Smoking			
yes	5	33	p=0.869;<br>1.212 (0.124-11.872)
no	1	8	
Drinking			
yes occasionally	6	25	P=0.156;<br>8.412 (0.444-159.497)
yes regularly	0	13	P=0.227;<br>0.162 (0.009-3.098)
no	0	3	p=0.915;<br>0.846 (0.039-18.367)
Clinical T- classification (T)			
T1	1	1	p=0.231;<br>6.000 (0.321-112.264)
T2	0	8	p=0.408;<br>0.285 (0.015-5.582)
T3	4	13	p=0.136;<br>4.000 (0.646-24.769)
T4	1	17	p=0.237;<br>0.259 (0.028-2.43)
NA	0	2	-
Nodal status (N)			
N0	4	25	p=0.903;<br>1.120 (0.182-6.907)
N1	0	5	p=0.636;<br>0.483 (0.024-9.828)
N2	2	7	p=0.400;<br>2.286 (0.347-15.042)
N3	0	2	p=0.929;<br>1.154 (0.050-26.887)
NA	0	2	-
Histological grading (G)			
G1	4	10	p=0.052;<br>6.200 (0.984-39.078)
G2	2	25	p=0.217;<br>0.320 (0.052-1.955)
G3	0	5	p=0.662;<br>0.511 (0.025-10.387)
G4	0	1	p=0.665;<br>2.077 (0.076-56.654)

OR- odds ratio; 95% CI - 95% confidence interval; NA – not assessed.

Table S9

Parameter	HPV(+)	HPV(-)	p-value; OR (95% CI)
Age (median (range))	65 (54-75.5)	66 (60-70)	p=0.972;
Sex			
female	1	11	p=0.410;<br>0.364 (0.033-4.035)
male	3	12	
Smoking			
yes	2	19	p=0.172;<br>0.211 (0.023-1.972)
no	2	4	
Drinking			
yes occasionally	3	18	p=0.895;<br>0.833 (0.070-9.858)
yes regularly	0	4	p=0.644;<br>0.482 (0.022-10.649)
no	1	1	p=0.196;<br>7.333 (0.357-150.717)
Clinical T- classification (T)			
T1	0	1	p=0.807;<br>1.519 (0.053-43.689)
T2	0	2	p=0.930;<br>0.867 (0.035-21.363)
T3	1	8	p=0.621;<br>0.542 (0.048-6.144)
T4	3	10	p=0.334;<br>3.300 (0.294-37.105)
NA	0	2	-
Nodal status (N)			
N0	3	12	p=0.512;<br>2.250 (0.200-25.370)
N1	0	3	p=0.740;<br>0.587 (0.026-13.531)
N2	1	4	p=0.786;<br>1.417 (0.115-17.461)
N3	0	2	p=0.930;<br>0.867 (0.035-21.363)
NA	0	2	-
Histological grading (G)			
G1	0	6	p=0.439;<br>0.299 (0.014-6.363)
G2	4	12	p=0.171;<br>8.280 (0.400-171.299)
G3	0	4	p=0.644;<br>0.482 (0.022-10.649)
G4	0	1	p=0.766;<br>1.667 (0.058-47.833)

OR- odds ratio; 95% CI - 95% confidence interval; NA – not assessed.

Table S10

Parameter	P16(+)	P16(-)	p-value; OR (95% CI)
Age (mean±SD)	64.75±16.01	63.76±11.16	p=0.873;
Sex			
female	6	11	p=0.381;<br>1.792 (0.486-6.615)
male	7	23	
Smoking			
yes	11	27	**p=0.014;**<br>**0.116 (0.021-0.651)**
no	7	2	
Drinking			
yes occasionally	10	21	p=0.332;<br>2.064 (0.477-8.920)
yes regularly	2	11	p=0.256;<br>0.380 (0.072-2.018)
no	1	2	p=0.821;<br>1.333 (0.111-16.090)
Clinical T- classification (T)			
T1	0	2	p=0.616;<br>0.452 (0.020-10.063)
T2	2	6	p=0.789;<br>0.788 (0.137-4.528)
T3	3	14	p=0.203;<br>0.386 (0.090-1.673)
T4	8	10	p=0.067;<br>3.520 (0.918-13.502)
NA	0	2	
Nodal status (N)			
N0	10	19	p=0.272;<br>2.281 (0.524-9.924)
N1	1	4	p=0.645;<br>0.583 (0.059-5.780)
N2	2	7	p=0.624;<br>0.649 (0.116-3.641)
N3	0	2	p=0.616;<br>0.452 (0.020-10.063)
NA	0	2	
Histological grading (G)			
G1	4	10	p=0.928;<br>1.067 (0.265-4.282)
G2	8	19	p=0.726;<br>1.263 (0.342-4.665)
G3	1	4	p=0.688;<br>0.625 (0.063-6.180)
G4	0	1	p=0.909;<br>0.827 (0.032-21.600)

OR- odds ratio; 95% CI - 95% confidence interval; NA – not assessed.
